# Focused deterrence intervention to reduce serious violence: study protocol for a randomised trial

**DOI:** 10.1186/s13063-026-09670-y

**Published:** 2026-04-07

**Authors:** Katharine A. Boyd, Neil Ralph, G. J. Melendez-Torres

**Affiliations:** 1 University of Exeter, Clayden Building, Streatham Campus, Exeter, EX4 4PE UK; 2 South West Police Collaboration, Devon & Cornwall Police HQ, Exeter, Middlemoor EX2 7HQ UK; 3 University of Exeter, South Cloisters, St Luke’s Campus, Exeter, EX1 2LU UK

**Keywords:** Focused deterrence, Crime prevention, Serious violence, Randomised controlled trial

## Abstract

**Background:**

Traditional policing interventions associated with the standard model of policing, such as programmes designed to arrest and prosecute repeat offenders, have not been effective in controlling crime. In contrast, a growing number of rigorous programme evaluations find focused deterrence strategies, designed to change offender behaviour through a blended law enforcement, social service and opportunity provision, and community-based action approach, are effective in controlling crime. While evidence is growing, there are few randomised controlled trials evaluating this type of intervention, particularly assessing the effect on repeat serious violence offenders.

**Methods:**

The police force will identify individuals involved in 3 or more serious violence offenses, with the most recent serious violence offense occurring within the last 24 months in the recorded crime data. Eligible individuals are placed within one of six strata based on gender and age group. Within each stratum, 50% of individuals are randomly allocated to the treatment condition and the rest to the control condition. All participants will receive usual care, but those randomly assigned to the experimental condition will receive a focused deterrence intervention visit from police providing a scripted empathetic talk and a list of local resources. The police will collect crime data for all individuals for the 12 months following the date of randomisation. The primary outcome is the total crime harm, measured by the Cambridge Crime Harm Index, perpetrated by the individual across all crimes committed in the year following randomisation (across England and Wales). The secondary outcomes include the number of arrests for violent crime, the total number of arrests, the number of non-violent arrests, and the time to the first arrest within the same timeframe.

**Discussion:**

Current RCTs investigating FD interventions focus on crime counts, but this assumes parity amongst crimes. Our study will assess the impact on both crime harm and crime counts to provide a comprehensive review of the impact of this intervention. If the hypotheses are supported, this single-contact FD intervention would likely have significant operational appeal for police and communities to prevent and reduce crime and harm with light-touch engagement.

**Trial registration:**

The study is now listed on the ISRCTN registry with study registration number ISRCTN35233331. Registered on 24 July 2024.

## Background and rationale {6a}

Reducing serious violence is a key objective of the National Police Chiefs Council (NPCC) 2030 vision. A growing number of rigorous programme evaluations find focused deterrence interventions to be an effective crime prevention strategy [3]. Focussed deterrence (FD) builds on theoretical insights on the proper application of deterrence principles to modify the behaviour of individuals and groups of high-rate offenders [9, 10]. Focused deterrence is a framework designed to change offender behaviour through a blended law enforcement, social service and opportunity provision, and community-based action approach, which are shown to be effective in controlling crime [4, 17]. Direct communication with offenders is a core component of FD strategies and is typically structured to include information about legal risks (sometimes tailored to the individual’s circumstances), service and support opportunities to encourage positive change, messages reflecting relevant community norms, and communication delivery aligned with procedural justice principles ensuring fairness and legitimacy in interactions.

However, despite growing support among law enforcement communities, randomised controlled trial (RCT) evidence remains thin with only five completed RCTs assessing focused deterrence strategies, only two of which have been published [1, 6].

Ariel, Englefield and Denley [1] found focused deterrence interventions with prolific offenders led to a crime suppression effect for that individual, as well as their co-offenders and the larger network (though to a lesser effect). Hamilton, Rosenfeld, and Levin [6] compared ‘intent-to-treat’ (ITT), the ‘average treatment effect’ (ATE), and ‘local average treatment effect’ (LATE) analyses to assess the self-selection bias for parolees and probationers attending a focused deterrence meeting on their subsequent criminal offending. Their results showed those who attended the FD meeting were less likely to be arrested in the next 17 months, and selection effects did not significantly impact the results.

There is an ongoing RCT (registered ISRCTN 11650008 by Prof. Brennan et al.; see [14]) investigating FD impact to reduce violent offending among 14–40-year-old individuals who have a previous involvement in violence. The study aims to assess the impact of the intervention on the number of violent offences in the year following.

Consequently, there is still a need for a trial to determine if a light-touch FD intervention can have a significant impact on behaviour to reduce crime harm among repeat violent offenders. The crime count minimises the different levels of physical and/or psychological violence associated with crimes and a measurement of crime harm, such as the Cambridge Crime Harm Index (CHI), is a useful tool for assessing the impact FD may have on the degree of harm perpetrated in subsequent criminal events.

Unlike traditional focused deterrence (FD) models that involve ongoing engagement and community representation, our intervention is distinct in its design: it consists of a single, structured meeting where police officers signpost individuals to community-based resources, allowing voluntary follow-up. This study protocol describes the methodology we are using to test a light-touch, single-contact focused deterrence (FD) initiative with perpetrators of serious violence, excluding domestic abuse (DA). The aim of the study is to identify if crime harm and subsequent offending can be reduced through the introduction of one-time FD visits, with the ability to signpost repeat violent offenders towards partner agency support.

### Objectives {7}

The aim of this study is to identify if crime harm and violent offending can be reduced with the introduction of single-contact focussed deterrence visits, with the ability to signpost people toward partner agency support.

We hypothesise that individuals who received the FD treatment will have lower crime harm (measured by the Cambridge Crime Harm Index for each offence) totalled in the following 12 months.

We also hypothesise that individuals in the treatment group will have fewer numbers of arrests for our secondary outcomes (violent crime arrests, total arrests, and non-violent arrests within the 12 months following randomisation) than individuals in the control group. Lastly, we hypothesise that the time to the first offence will be greater for individuals receiving the FD intervention than individuals in the control group.

### Trial design {8}

This is the protocol for a superiority randomised controlled trial with a two-group parallel design with units randomised on a rolling basis.^1^ The unit of randomisation is the serious violent offender and the allocation ratio between the control and intervention groups is 1:1.

## Methods: participants, interventions, and outcomes

### Study setting {9}

This trial takes place in Devon & Cornwall Constabulary, one of the 43 police force areas in England and Wales. Integrated Offender Management (IOM) officers, already deployed across the force area, will conduct FD visits to the homes of known repeat violent offenders assigned to the experimental condition.

### Eligibility criteria {10}

The police force will identify individuals for this study in the recorded crime data. To be eligible for inclusion in the study, individuals must fulfil the following inclusion criteria:

1. Identified in police data as being involved in 3 or more serious violence offenses
2. The most recent serious violence offense must be within the last 24 months

For the purposes of this study, serious violence was defined subjectively as any offence at the level of actual bodily harm (ABH) or above, including assaults on police officers or emergency services workers. See the Appendix for a list of all serious violence crimes used for inclusion criteria.

Individuals are not included in the sample if any of the following exclusion criteria are identified:

1. Have a record of a domestic abuse violent crime arrest
2. In prison at the time of randomisation
3. Identified as out of the country at the time of randomisation
4. Assigned to Multi-Agency Public Protection Arrangements (MAPPA)
5. Assigned to Management of Sexual or Violent Offenders (MOSOVO).

### Who will take informed consent? {26a}

Informed consent is not obtained for this study because data is not being gathered from the participants directly. Interaction with the participants involves the IOM officer speaking to participants and giving them a list of partner support agency resources. The interaction does not require the participant to provide any data or information. Instead, data about the participants’ subsequent criminal activity, data which belong to the police force, will be used for outcomes.

### Additional consent provisions for collection and use of participant data and biological specimens {26b}

This trial does not involve informed consent. Data will only be used for the purposes outlined in the protocol. If there are any plans for ancillary studies using the data, including for data management purposes, we will obtain additional approvals from the ethics committee and data controllers, and ensure consent is obtained, if needed, prior to undertaking such work.

## Interventions

### Explanation for the choice of comparators {6b}

Individuals assigned to the control group will not receive the FD treatment. These individuals will receive business as usual, meaning these individuals are dealt with through the standard criminal justice process, which does not include doorstep follow-up or additional community-based intervention. These individuals experience routine procedures independent of the intervention, which may include receiving care from the police, Youth Offender Team (YOT) members, and other social affiliates as deemed necessary.

### Intervention description {11a}

The FD intervention incorporates the key elements of FD communication. IOM officers visit the home of individuals in the treatment group and give a scripted talk to show empathy and concern for the participant’s future health and safety and provide the participant with a list of partner agency support resources available in the individual’s area. The intervention is grounded in the principles of procedural justice, with officers trained to exhibit empathy, kindness and compassion using a trauma-informed approach that considers Adverse Childhood Experiences. Specifically, IOM officers are instructed to:

- Confirm name of subject
- If juvenile—ensure a suitable adult is present
- Introduce self—name and role
- Explain that this visit is separate to any other programme of supervision they may be under
- Explain they are NOT in trouble with the police
- Explain the reason for the visit is that the subject has been identified as someone who has been arrested or is shown as a suspect 3 times for offences of violence in a 24-month period
- Explain the reason for the visit is to deter them from being involved in violent offences in the future
- Be empathetic. Explain we are really concerned that they will get seriously injured, or will seriously injure someone else, which could result in a prison sentence. What impact would either of these things have on the subject, their family, their friends, their loved ones?
- If possible, a discussion around why they have been involved in acts of violence. What could they do to avoid these situations in the future?
- Introduce the signposting leaflet. Leave with the subject. Explain that some of the agencies are national, some are local. Some may be relevant to the subject, some may not. But they should consider contacting any agencies that the subject believes could offer them support to avoid offending in the future
- Finish by explaining Devon & Cornwall Police as an organisation are really concerned about the subject and that we want to help them. We want to avoid them being in violent situations in the future and we want to keep local communities and themselves safe—hence the reason for the visit
- Devon & Cornwall Police have a positive obligation to safeguard and protect communities—so if they commit further violent offences they may be arrested and could, in addition to a criminal investigation, be subject to civil orders and a criminal behaviour order
- Finish and leave on good terms

The IOM officers will review each individual’s profile with all known intelligence in real time before approaching the individual’s home. These officers’ training is up to date, and they are equipped to manage interactions with violent offenders as part of standard practice.

The IOM officers will make 3 attempts to visit with individuals in the experimental condition. If the participant is not home on the third attempt, the officer will leave a document with a focussed deterrence statement and the list of partner agency support resources. The individuals will be analysed based on the intention-to-treat as they will be given the FD document if they are in the treatment group and do not answer the door.

IOM officers will record implementation data based on their impression of the individual’s level of cooperation (i.e. Likert scale: 1—very uncooperative to 5—very cooperative) and a tick box indicating if anyone else was at the home during the visit (i.e. spouse, partner, sibling, mother, father, guardian, friend).

### Criteria for discontinuing or modifying allocated interventions {11b}

The discontinuation of an individual in the trial would only occur if the IOM officer deemed the risk of making the FD intervention too great to proceed. If this is due to environmental factors that may change at another time, the intervention would be attempted on another day. If the intelligence gathered suggests the risk is too great, then the individual would be discontinued from the intervention.

### Strategies to improve adherence to interventions {11c}

The intervention period is very short and IOM officers will be contacted by members of the research team on a monthly basis to improve adherence to the intervention protocol.

### Relevant concomitant care permitted or prohibited during the trial {11d}

Individuals in the control group will not receive the FD intervention, but all other care or interaction with police or other social services is permitted during the trial.

### Provisions for post-trial care {30}

Individuals in both conditions will not receive any ancillary or post-trial care beyond what is normally provided by the criminal justice and social service systems.

### Outcomes {12}

The primary outcome is the total crime harm (domain) perpetrated by the individual across all crimes (based on official arrest reports across England and Wales^2^) committed in the 12 months following randomisation (time point). Crime harm is measured by the Cambridge Crime Harm Index (CHI) (measure).^3^ This value is meant to capture the relative difference in harm between offenses, giving violent offenses a higher CHI score than non-violent offenses. The CHI score is the number of days incarceration recommended for each offense in the National Sentencing Guidelines in England and Wales [12, 13]. We will compare the cumulative Cambridge Crime Harm Index (CHI) score (method of aggregation) across all crimes for which individuals are arrested in the 12 months following randomisation, between the intervention and control groups (metric). We hypothesise that individuals who received the FD will have a lower cumulative CHI score than those in the control group.

The secondary outcomes are: (1) number of arrests for violent crime, (2) total number of arrests, (3) number of non-violent arrests, and (4) time to first offence. All outcomes are based on official crime reports across England and Wales (measure) within 12 months following randomisation (time point).^4^ For outcomes 1–3, the domain is arrests, the method of aggregation is count per individual, and the metric is the difference between intervention and control groups at 12 months. For outcome 4, the domain is time to first offence, measured in days from randomisation, aggregated as time-to-event, with the metric being the difference between groups at the 12-month time point.

### Participant timeline {13}

Individuals meeting the inclusion criteria are identified by the police force. Eligible individuals are placed within one of six strata based on gender and age group. Within each stratum, 50% of individuals are randomly allocated to the treatment condition and the rest to the control condition.

Because of the trial timeline, we anticipated that we would have a large sample size identified at baseline, with additional individuals accumulated over time. This created a question of how to manage the large sample size at baseline in light of operational constraints. In consultation with operational colleagues, we identified that ‘enrolling’ participants in batches of 40 was optimal. To do this, we will randomly assign individuals into batches of 40, with 20 individuals in each condition, stratified by both trial arm and randomisation strata (i.e. 12 strata overall). Each batch of 40 is then sent to the police sequentially, with the date of transmission marked as the date of randomisation (i.e. starting the clock for the 12-month follow-up period). Following on from this, the IOM team will complete the FD interventions for the treatment group as quickly as possible. The police will collect crime data for all individuals for the 12 months following the date of randomisation.

### Sample size {14}

We intend to recruit until we get to a sample of 300 individuals to ensure statistical power for the analyses. This sample size generates just over 90% power to detect a standardised effect on the primary outcome of 0.4, using Julious’ [8] standard formulae:

$$n=\frac{2{\sigma }^{2}({Z}_{1-\alpha /2}+{Z}_{1-\beta }{)}^{2}}{{\delta }^{2}}$$

where:

- $n$ = sample size per group
- $${\sigma }^{2}$$ = variance
- $\delta$ = expected difference between groups
- ${Z}_{1-\alpha /2}$ and ${Z}_{1-\beta }$ = standard normal values for significance level and power

### Recruitment {15}

Individuals are not recruited as such for this study, but are identified by meeting the inclusion and exclusion requirements in police data.

## Assignment of interventions: allocation

### Sequence generation {16a}

We will generate six strata by gender (male; female) and age group (15–17; 18–24; and 25+ years old).^5^ Once individuals are stratified, we will use computer-generated random numbers via the Stata programme -ralloc- to conduct randomisation to the experimental or control condition with a 1:1 ratio and varying block size within stratum.

## Concealment mechanism {16b}

Randomisation is undertaken by the trial statistician (GJMT) separate from the delivery team (led by NR) and the chief investigator of the trial (KB). Individuals are randomised in batches of 40 (20 in the experimental group and 20 in the control group). IOM officers who will carry out the intervention only receive information for the individuals allocated to the experimental group and do not have access to the full randomisation list, the sampling frame, who has yet to be allocated, or details of participants in the control group. The team responsible for randomisation, data management, and analysis is separate from those delivering the intervention to ensure allocation remains concealed and minimise risk of bias. The date of randomisation for each individual starts when the batch has been made known to the IOM officers, soon after which those in the experimental group will receive the intervention.

### Implementation {16c}

The trial statistician will generate the allocation sequence and assign participants to the control or experimental condition in bulk at baseline and subsequently in batches of 40. Randomisation batches will then be given to the IOM officers on a rolling basis.

## Assignment of interventions: blinding

### Who will be blinded {17a}

Due to the type of intervention, providers and recipients will not be blinded in this trial. The IOM officers need to know which individuals are in the intervention group to know who receives the FD intervention. The trial statistician will be blinded to assignment at the point of analysis. Outcome data will be collated by analysts in the police force separate from the delivery team, meaning outcome assessors will be blinded as well. The chief investigator will hold the ‘unblinding code’ and will reveal group assignments once analysis by the trial statistician is complete.

### Procedure for unblinding if needed {17b}

Not applicable as no blinding was used in this trial.

## Data collection and management

### Plans for assessment and collection of outcomes {18a}

The police force will provide the crime data that will be used for eligibility in the trial, and then for the outcome measures. This will be provided by analysts blinded to treatment allocation. The IOM officers will collect implementation data as a part of their follow-up report. The IOM officers will have been instructed how to complete this data collection properly.

### Plans to promote participant retention and complete follow-up {18b}

Owing to the type of intervention and outcome data that is collected by authorities as a part of their profession, there are no plans for participant retention or follow-up.

### Data management {19}

The crime data is collected by the police force and stored in secure data storage meeting legal standards. Offense records from the Police National Computer (PNC) or its forthcoming replacement, the Law Enforcement Data Service (LEDS), will also be used to capture data nationally so subsequent arrests and offenses between districts will be included. It will be de-identified by the police force, which is then shared with the research team and stored on a secure SharePoint site hosted by the university that is backed up daily. The chief investigator of the trial will be responsible for managing the transfer of data and will ‘blind’ the dataset to treatment allocation at the point of preparation for analysis. The implementation data will be collected by IOM officers, collated by the police force, and stored in their data storage. A separate table of characteristics of the study population will be prepared in summary format by the force to assist reporting but will not be linked to outcome data. Any potentially disclosive data will be removed before reporting.

### Confidentiality {27}

The crime data is collected by the police force as part of normal operations, which includes personal and identifying information as needed for legal purposes. However, for this study the police will create a unique ID for each participant in the study that will remain linked to the participant crime data recorded in the following 12 months. Only this de-identified data required for analysis will be shared with the research team to maintain confidentiality.

### Plans for collection, laboratory evaluation and storage of biological specimens for genetic or molecular analysis in this trial/future use {33}

Not applicable as this trial does not involve collection of any biological specimens.

## Statistical methods

### Statistical methods for primary and secondary outcomes {20a}

Because of the limited number of outcomes collected, the statistical analysis plan has been included in the text as below.

The primary and secondary outcomes will be evaluated in an analysis of covariance (ANCOVA) framework using intention to treat (ITT) principles.^6^ A nominal significance level of 0.05 with two-sided tests will be used for all analyses.

All analysis models will include the outcome as a dependent variable, with regression terms for intervention allocation, stratum, round of randomisation, and whether the most recent arrest was less than a year from randomisation.^7^ The choice of link function will be based on the outcome distribution.

For the primary outcome (Cambridge Crime Harm Index), an identity link will be preferred. The CHI often has a highly skewed distribution, making standard linear modelling problematic. If preliminary checks suggest that normality is likely to be severely violated, confidence intervals will be generated using a bias-corrected and accelerated bootstrapping method with 10,000 draws.

For the primary outcome of CHI, the equation is:

$$E({Y}_{i})={\beta }_{0}+{\beta }_{1}{trt}_{i}+{X}_{i}{B}_{2}+{{\beta }_{3}{arrest}_{i}+Z}_{i}{B}_{4}$$

where *Y*_*i*_ is the outcome for a participant *i*, *trt*_*i*_ is the intervention group indicator, *X*_*i*_ is the vector of randomisation strata defined by age and gender, with any one participant recording 1 for the relevant stratum and 0 for all others (males aged 18–24 as the reference category), and *Z*_*i*_ is the vector of randomisation rounds, with any one participant recording 1 for the relevant round and 0 for all others (the first randomisation round as the reference category), and *arrest*_*i*_ is a binary variable indicating arrest in the 12 months prior to randomisation.

For the outcomes of the number of arrests and the number of arrests for violent crime, a log link with a Poisson distribution will be used, with an exposure variable linked to follow-up time.

For a count-distributed variable, the model is:

$${E(\mathrm{ln}(Y}_{i}))={\beta }_{0}+{\beta }_{1}{trt}_{i}+{X}_{i}{B}_{2}+{{\beta }_{3}{arrest}_{i}+Z}_{i}{B}_{4}+{offset}_{i}$$

reflecting the use of a log link. An offset for time is defined as the log of the follow-up time with the coefficient constrained to 1.

For the outcome of time to first arrest, a Cox proportional hazards model will be used. Participants will be right-censored at 12 months if no arrest has been recorded over the follow-up period. The model for this outcome is:

$$E(lnHR)={\beta }_{1}{trt}_{i}+{X}_{i}{B}_{2}+{{\beta }_{3}{arrest}_{i}+Z}_{i}{B}_{4}+{offset}_{i}$$

### Interim analyses {21b}

We do not plan for an interim analysis.

### Methods for additional analyses (e.g. subgroup analyses) {20b}

We will examine effect modification by stratifying variables (age group, gender) and by celerity (whether the last arrest was 12 months or more from the date of randomisation), as well as by the count of violent offences in the 24 months prior to randomisation. In addition to descriptive statistics for sub-groups to provide context, we will conduct exploratory effect modification analyses that will use a standard likelihood ratio test comparing models with interaction terms against models without interaction terms for each moderator separately, and will be undertaken for all outcomes. As in main effects models, all regressions will include terms for intervention allocation, strata, round of randomisation, and celerity.

Following Francis et al.’s ([5, 16, 17]) suggestion, we will assess exploratory outcomes by categorising individuals based on harm and frequency into three groups: *serious but not frequent*, *frequent but not serious*, and *frequent and serious* within the 12 months following randomisation. This will allow us to assess whether the intervention has impact on harm in relation to frequency.

Finally, we will explore sensitivity of the analysis of the primary outcome to choice of regression link. We will use exploratory specifications of the analysis model using a Poisson regression with robust standard errors, and using a semiparametric ordinal regression model [7] to calculate mean ratios and proportional odds ratios correspondingly.

### Methods in analysis to handle protocol non-adherence and any statistical methods to handle missing data {20c}

Because all outcome data are routinely collected, we do not expect to have high levels, if any, missingness with respect to our outcomes. However, some missingness might arise from intercurrent events such as death or long-term departure from the country that would mean they are not ‘at risk’ for any of the trial outcomes. From pilot data, we expect these to be very rare events over the follow-up period and unlikely to be related to intervention allocation. If any participants experience these ‘competing risks’, then we will censor participants at that time point. We will use crime harm index scores accumulated to that point and use the time from randomisation to point of censoring as the value for exposure. We will review the data to assess cases with missing data or extreme (outlier) values to ensure accuracy.

We will conduct exploratory complier adjusted causal effect (CACE) analysis for the primary outcome to account for non-adherence or non-receipt of the intervention. If compliance is complete, this analysis will not be necessary, but otherwise it will be implemented using an instrumental variable (IV) approach [2]. Using intervention allocation as the instrument, receipt of the intervention as the mediating variable, and strata indicators as additional covariates, we will test whether receipt of the intervention (as opposed to assignment to the intervention) causes a decrease in crime harm caused.

### Plans to give access to the full protocol, participant-level data and statistical code {31c}

We will provide the full protocol and statistical code when the study has been published; however, participant-level data will not be available.

## Oversight and monitoring

### Composition of the coordinating centre and trial steering committee {5d}

Members of the police force operate as the coordinating centre. These members have varying responsibilities: those working with the crime data and those assessing individual risk, the IOM officer leads coordinating the FD intervention visits, and the Detective Chief Superintendent (author) coordinates these activities with external academics on the research team.

The Trial Steering Committee includes three external individuals including expertise from academia, the College of Policing, and from operational policing. The Trial Steering Committee will meet periodically over the course of the trial.

### Composition of the data monitoring committee, its role and reporting structure {21a}

The Trial Steering Committee will address any data monitoring functions.

### Adverse event reporting and harms {22}

We convened our Trial Steering Committee to discuss potential harms and mitigation strategies. To guide this process, we developed a dark logic model to theorise how and why the intervention could produce harm, and whether certain individuals or groups might experience disproportionate harm, applying an equity lens as suggested by Li et al. [11].

Several potential harms were considered:

- Whether the intervention could have unintended effects increasing rather than reducing offending.
- The possibility that serious violent offences or other crimes might occur if individuals misinterpret the visit (e.g. suspecting someone ‘grassed them up’).
- The risk of stigmatisation for those visited by police. We believe this risk is mitigated using plain-clothes officers and the supportive tone of the intervention.

It is possible an IOM officer will be told information about intended harm to the participant or another individual when conducting FD interventions. The IOM officers are trained to intervene appropriately to address risk as part of their standard duties. Any information disclosed or harm experienced would be written up in the IOM report that is then within police data systems. All harms, whether anticipated or unexpected, will be reported in the final trial report for transparency.

### Frequency and plans for auditing trial conduct {23}

This is a small single-centre, data-enabled trial. The investigator team will receive monthly reports throughout the intervention delivery period tracking intervention fidelity and delivery. The Trial Steering Committee will periodically receive updates on trial conduct.

### Plans for communicating important protocol amendments to relevant parties (e.g. trial participants, ethical committees) {25}

The coordinating centre for the trial will distribute any protocol changes that will impact how the study is conducted to all relevant parties. These include the Ethics Committee of HASS at the University of Exeter, the IOM officers in the force area, police staff managing crime data, the academics, the Trial Steering Committee, as well as updating the registration of the trial with ISRCTN.

### Dissemination plans {31a}

The trial results will be published in a peer-reviewed criminology journal as well as disseminated at both national and international criminology meetings. We will also produce a report with results of the study for public consumption.

## Discussion

The existing empirical evidence suggests that ‘person-focused’ policing interventions associated with the standard model of policing, such as programmes designed to arrest and prosecute repeat offenders, have not been effective in controlling crime [15]. In contrast, a growing number of rigorous programme evaluations suggest that FD strategies, designed to change offender behaviour through a blended law enforcement, social service and opportunity provision, and community-based action approach, are effective in controlling crime [17, 4]. This trial will contribute to this growing literature by focusing on repeat serious violence offenders and considering the impact of a single-contact FD intervention on the cumulative crime harm in the year following. Current RCTs investigating FD interventions focus on crime counts, but this assumes parity amongst crimes. Our study will assess the impact on both crime harm and crime counts to provide a comprehensive review of the impact of this intervention. If the hypotheses are supported, this single, one-time FD intervention, a departure from the classic FD framework, would likely have significant operational appeal for police and communities to prevent and reduce crime and harm with light-touch engagement requiring little additional effort or resources.

## Trial status

This study was approved by the Ethics Committee of University of Exeter (Ethics application ID: 6054510) on 5th April 2024. The study was registered with ISRCTN (study registration: ISRCTN35233331, Protocol Version 21.6.2024 v1). The first group of individuals was randomised to conditions on 8th May 2024. Recruitment is complete in March 2025. See the SPIRIT figure below.

**Table:** 

	**Enrolment**	**Allocation**	**Post-allocation**	**Close-out**
**Timepoint**	*−t*_*1*_ = Started April 2024 and is ongoing with expected completion on 1st October 2024	0 = units randomised on a rolling basis starting 8th May 2024	*t*_*1*_ = Intervention is completed as soon as possible following allocation	*t*_*x*_ = 12 months following randomisation
**Enrolment**:				
Eligibility screen	X			
Allocation		X		
**Interventions**:				
Focused deterrence visit			X	
**Assessments**:				
Primary outcome^a^: total crime harm				X
Secondary outcomes^a^: • Number of arrests for violent crime Total number of arrests, • Number of non-violent arrests • Time to the first arrest				X

^a^The outcomes are for all crimes (based on official reports across England and Wales) perpetrated by the individual across committed in the 12 months following randomisation

## Data Availability

The police force will have access to the final trial dataset, including the implementation data collected by IOM officers. The de-identified data will be shared with the research team for analysis purposes using a secure SharePoint site hosted by the university.

## Appendix

Serious violence crimes used for the inclusion criteria.

**Table:**

Offence description	Offence type
Manslaughter (recordable)	Homicide
Murder—victim 1 year of age or older	Homicide
Section 18—grievous bodily harm with intent (recordable)	Violence with injury
Attempt to cause grievous bodily harm with intent to do grievous bodily harm (recordable)	Violence with injury
Wound/inflict grievous bodily harm without intent (recordable)	Violence with injury
Racially/religiously aggravated assault occasioning actual bodily harm (recordable)	Violence with injury
Section 18—wounding with intent (recordable)	Violence with injury
Racially/religiously aggravated wounding/grievous bodily harm (recordable)	Violence with injury
Attempt murder—victim aged 1 year or over (recordable)	Violence with injury
Non-fatal strangulation and suffocation—Serious Crime Act 2015 s75	Violence with injury
Racially aggravated assault/actual bodily harm (recordable)	Violence with injury
Section 18—attempt wounding with intent (recordable)	Violence with injury
Possess a firearm with intent to endanger life/enable another to do so (recordable)	Violence with injury
Inflict grievous bodily harm without intent (Section 20)	Violence with injury
Attempt to wound/cause grievous bodily harm without intent (recordable)	Violence with injury
Make use/attempt to make use of a firearm with intent to resist arrest (recordable)	Violence with injury
Racially or religiously aggravated assault occasioning actual bodily harm (Sec 47)	Violence with injury
Attempt murder	Violence with injury
Choke/suffocate/strangle to render unconscious/incapable of resistance with intent to commit indictable offence (recordable)	Violence with injury
Racially or religiously aggravated wounding/grievous bodily harm (Sec 20)	Violence with injury
GBH/Wounding on a Constable (recordable)	Violence with injury
GBH/Wounding with intent on a Constable (recordable)	Violence with injury
Section 18—cause grievous bodily harm with intent to resist/prevent arrest (recordable)	Violence with injury
Possess shotgun with intent to endanger life/enable another to do so (recordable)	Violence with injury
GBH/wounding on an emergency worker (recordable)	Violence with injury
Cause administer poison/noxious thing with intent to injure/aggrieve/annoy (recordable)	Violence with injury
Assault occasioning actual bodily harm (Section 47)	Violence with injury
Assault a person thereby occasioning them actual bodily harm (recordable)	Violence with injury
Assault with intent to resist arrest (recordable)	Violence with injury
Robbery (recordable)	Robbery
Attempt robbery (recordable)	Robbery
Assault with intent to commit robbery (recordable)	Robbery
Robbery—personal (recordable)	Robbery
Assault with intent to commit robbery—personal (recordable)	Robbery
Assault with intent to commit robbery—business (recordable)	Robbery
Attempt burglary dwelling with intent to inflict grievous bodily harm (recordable)	Burglary—residential
Burglary dwelling—with intent to inflict grievous bodily harm (recordable)	Burglary—residential

## Abbreviations

ATE: Average treatment effect
CHI: Crime Harm Index
FD: Focused deterrence
IOM: Integrated Offender Management
ITT: Intent-to-treat
LATE: Local average treatment effect
RCT: Randomised controlled trial

## Footnotes

1 This study was designed as a definitive evaluation of a light-touch intervention rather than as a pilot or feasibility trial. It does not include feasibility endpoints or progression criteria characteristic of a pilot design.

2 Arrest is an upstream measure in the criminal justice process. In comparison, charge, caution or conviction of a crime are criminal justice outcomes that indicate investigation has taken place. However, we have retained arrest for our outcomes because it provides a timely and practical indicator of subsequent offending within the 12-month follow-up period. Charge, caution or conviction as outcomes introduce significant delays due to the investigative and court processes, which often extend well beyond one year. Arrest is not a perfect measure, but it is the most feasible option for this study design. Future research should seek to explore these longer-term outcomes for light touch FD interventions.

3 Measuring the seriousness or harm of crime presents significant challenges, and there is no universally agreed-upon methodology (see Sutherland, 2013). Approaches such as the Cambridge Crime Harm Index (CHI) offer one way to quantify harm, but like other measures, CHI has limitations and relies on assumptions that may not fully capture the complexity of offending. We acknowledge these difficulties and reference existing discussions on measurement approaches. To provide additional context, we include different forms of incident frequency as secondary outcomes for comparison.

4 The classifications of ‘violent crime’ (secondary outcome 1) and ‘non-violent crime’ (secondary outcome 3) correspond to the police-recorded crime categories (with violent crime aligning to the ‘violence against the person’ category). These differ from the serious offences specified in the study’s inclusion criteria, which are provided in the Appendix.

5 We did not stratify the sample by individual’s ethnicity because this region of the country has limited ethnic diversity. However, we will obtain ethnicity data for all individuals in both the intervention and control groups to assess whether any ethnic group was disproportionately allocated to one condition.

6 The ITT population includes all individuals who were identified as eligible and subsequently randomized, regardless of attendance, completion, or participation. No exclusions were made on the basis of non-completion or non-participation.

7 Due to the availability of data, we are unable to include a measure of group or gang -involved activity. While group involvement and associated dynamics may influence outcomes, this data is not available, but future research should explore the role of group dynamics in light touch, single-contact FD interventions.
